# Women’s education level amplifies the effects of a livelihoods-based intervention on household wealth, child diet, and child growth in rural Nepal

**DOI:** 10.1186/s12939-017-0681-0

**Published:** 2017-10-18

**Authors:** Laurie C. Miller, Neena Joshi, Mahendra Lohani, Beatrice Rogers, Shubh Mahato, Shibani Ghosh, Patrick Webb

**Affiliations:** 1 Department of Pediatrics, Tufts University, Boston, MA USA; 2 Heifer Nepal, Kathmandu, Nepal; 3 Heifer International, Little Rock, AR USA; 4 Friedman School of Nutrition Science and Policy (FSNSP), Tufts University, Boston, MA USA

## Abstract

**Background:**

Many organizations seek to alleviate poverty in the developing world, often focusing their interventions on women. The role, status, and education of women are fundamentally important facets of development. Thus, understanding the interaction of women’s educational level and the response to interventions is important. Therefore, we examined the impact of educational level of household adults on responses to a livestock-based community intervention.

**Methods:**

Six pair-matched communities in 3 districts of Nepal (Chitwan/Nawalparasi/Nuwakot), were randomly assigned to receive community development activities via women’s self-help groups at baseline or 1 year later. At 6 intervals over 48 months, a 125- item questionnaire addressing family demographics and child health/nutrition was completed in each household, plus child growth monitoring. Results were analyzed in relation to the highest education attained by *any* woman in the household, the child’s mother, men, or any other adult in the household.

**Results:**

Outcomes (wealth, water/toilet availability, child diet diversity and growth) all significantly related to adult education. However, notable differences were found comparing the impact of men’s and women’s education. Percent change in wealth score was significant only in households where women had primary or secondary education (respectively, *p* = .0009 and *p* < .0001). Increased soap use related only to women’s education (*p* < .0001). When adjusted for group assignment, baseline income, wealth, and animal scores, higher women’s education was significantly associated with increased household wealth (*p* < .0001), better child height-for-age z scores (HAZ, *p* = .005), and improved child diet diversity (*p* = .01). Higher mother’s education predicted better child HAZ (primary, *p* = .01, secondary, *p* = .03) and diet diversity (primary, *p* = .05, secondary, *p* < .0001). Higher men’s education was significantly associated with household wealth (*p* = .02) and child diet diversity (*p* = .04), but not HAZ; higher education of any household member was associated only with household wealth (*p* < .0001). Moreover, households where the mother’s education was better than the best-educated man also were significantly more likely to have children with better HAZ and dietary diversity (*p* = .03, *p* < .0001). Thus, the educational level of women and mothers had the broadest impact on child outcome variables.

**Conclusions:**

Household characteristics vary among participants in most community development projects. Of these, adult education likely mediates response to the inputs provided by the intervention. Particularly in interventions directed towards women, better education may enhance the ability of households to put interventions into practice, thus improving wealth, hygiene, and child diet and growth indices.

## Background

Integrated, multi-sectoral approaches to poverty in the developing world, although more complicated to implement, are more likely to result in sustained improvement in household and individual economic status than programs with a single focus. Such strategies have been the basis for many programs which seek to link agriculture-based interventions, household wealth, and child nutrition outcomes [1–4]. Although some successes have been reported, the links between household wealth and child growth status are not automatic [5]. At a national level, marked differences exist between the prevalence of stunting and GDP [6]; at the household level, a complex relationship exists between income and child nutritional outcomes [7, 8]. Although improved socioeconomic status could improve nutritional status by creating a healthier environment (with more access to medical care, clean water, and sanitation [2, 6, 9–11]) or more diverse diet [12], increased household income may not always be directed toward child health needs or providing optimal foods for children [3]. Some of these differences may be explained by gender empowerment, behavior change (at the community and household level), and control over household resources.

Previously, we evaluated the impact of a livestock-based livelihoods intervention on child growth and diet outcomes [13–15]. The intervention, conducted by *Heifer International Nepa* *l*, consisted of a strong social capital development program, but without a specific nutrition or health focus or *any* emphasis on child-specific outcomes. In the initial analysis of this dataset, duration of exposure to the Heifer intervention was associated with improved household wealth, child growth, and child diet . However, more detailed analysis at the household level revealed a range of different outcomes: some families benefitted greatly while the conditions of other families did not improve, or even worsened. This finding prompted our search for an explanation. Recent findings highlight that the effectiveness of interventions depends a great deal on household-level exposure to and uptake of the goods and services on offer to them [16]. The degree of uptake is mediated by a wide range of household and individual characteristics, including baseline wealth, sources of income, demographics, and extent of participation, among others [2, 17, 18]. We sought a reasonable explanation for the discrepant outcomes by examining the impact of the wide range of educational achievements of women in our project area.

A key element much discussed in the existing literature is the role of gender empowerment. While level of household income matters, how and by whom it is used can matter more. While technologies may offer enhanced productivity in agriculture, if they cannot be used by women who are heavily engaged in agriculture, then adoption of these practices may be limited. In any case, the impact on children’s well-being may be less if women are not involved. In other words, the role, status, knowledge and education of women in the household are fundamentally important facets of development, and often a deciding factor in the success or failure of programs supporting improved diets and nutrition for children [6, 19]. In addition, many programs focus their intervention efforts on women. Thus, understanding the interaction of women’s educational level and the response to the intervention is important. The relative lack of attention to women’s educational level and intervention outcomes is surprising, given the growing international focus on the importance of female education [20, 21].

Maternal education affects the well-being of children in many ways, specifically in protecting against mortality [11, 22], improving child health [23], development [24], and growth (height) [6, 10, 25]. However, little is known about how women’s educational attainments – or that of other members of the family - may influence household responses to community-level interventions over time. We hypothesized that the level of educational attainment by adults in the household, particularly of women, predicted longitudinal changes in specific household variables (wealth and income, household amenities and hygiene practices) and important child outcomes (health, growth, and dietary diversity). The goal was to identify characteristics of women and their households (including the educational attainments of other family members) conducive to successful program outcomes.

## Methods

### Ethics, consent and permissions

This study was approved by the Nepal Health Research Council (Reference Numbers 845 and 901) and Tufts University. Consent was obtained from participants at each visit in accordance with practices approved by these human investigation review boards.

### Setting and study design

This 48 month longitudinal randomized controlled trial was conducted in 3 districts of Nepal --Chitwan and Nawalparasi [Terai], Nuwakot [hills]. The intervention was implemented by *Heifer International*, an international non-governmental organization (NGO) which seeks to eliminate poverty via livestock-based community development programs. Services and inputs are provided to targeted communities at the request of local NGOs, which interact with Village Development Committees (VDCs). For purposes of this study, 3 pairs of communities were identified in each district, matched based on geographic location (including altitude), size of population, type of natural resources, local employment opportunities, health care facilities, type of agriculture, and other socioeconomic features such as caste, income and education level. Known local health risks (e.g. lead exposure, iodine deficiency) were also taken into account in the matching process. In each community, local leaders served on an advisory panel and as liaisons to the population about the project activities. Families within each community were invited to participate. *Heifer International* attempts to include all households for participation in the intervention: participation rates exceeded 90% of households in each study site.

A staggered intervention design was used (previously described in detail [13–15]. The paired communities were randomly assigned to receive Heifer development activities either immediately (Group 1) or 12 months later (Group 2). Data collection was conducted at baseline, every six months through 24 months, and again at 48 months. Thus, Heifer activities began in Group 1 communities after the baseline survey; these activities continued through the entire 48 months. Heifer activities began in Group 2 communities 12 months later, continuing through to 48 months. Group 1 and Group 2 communities were non-adjacent to minimize “spill-over” effects.

Data collection was performed by a local field research NGO (Nepal Technical Assistance Group) not connected to Heifer. Field supervisors monitored the performance and activities of the Field Enumerators, and conducted daily reviews of the data collection to allow rapid identification and correction of errors and omissions. Enumerators were trained at the beginning of the project with 1 week of orientation to the project, followed by field pilot testing in 3 villages not included in the project sites, and ongoing quality control and refresher training activities to monitor and maintain inter-observer reliability. Enumerators were blinded to the assignment at baseline of Groups 1 and 2. At each field visit, enumerators completed a 116-item questionnaire with the female head of household (or her designee, in <3% of 2115 household interviews conducted). The questionnaire was based on standardized tools used in the Demographic and Health Survey conducted by the Government of Nepal (2007) [26]. At all 6 time points, data collection also included anthropometric measurements and health information on all enrolled children (described below).

### Intervention

The intervention consisted of an intensive 12 month program of participatory community development led by Heifer staff, focusing on tools for poverty alleviation (particularly via optimization of livestock management), citizen empowerment, and community development [27] These activities are based in women’s Self-Help groups, which meet weekly or biweekly with a trained facilitator to discuss local and personal issues in the context of values training, gender and family issues, social mobilization, group strengthening, microcredit, and enterprise development. At the conclusion of the 12 month curriculum, each participating household receives 2 meat-type goats. Notably, the Heifer training curriculum is not specifically individualized for participants with different educational backgrounds, and neither child diet nor household hygiene practices are specifically addressed.

### Participants

All members of each participating family were enrolled in the study. Children between ages 6 months and 8 years who resided in participating families were evaluated in more detail, with anthropometry, diet recall, and health survey. Child age was verified by inspection of the birth certificate or vaccine card. Children who “aged in” to the entry criteria were enrolled in the study at the first visit at which they were eligible. Children who “aged out” of the study were followed for the duration of the study. The targeted age for this study was children 6–60 months. Children with physical disabilities including blindness, deafness, inability to crawl or walk as appropriate for age, inability to communicate in an age-appropriate manner, obvious birth defects, or neurologic handicaps that prevented ingestion of a normal diet for age were excluded from enrollment. In addition, children with severe inter-current illnesses at the time of survey (e.g., high fever, severe diarrhea, or other symptoms which parents considered would make the child’s participation too difficult) were excluded from that round of data collection.

### Educational level of adults in households

At each visit, the educational attainments of all adult household members (over 15 years of age) were reviewed, and categorized as (1) none, (2) literacy classes/non-formal education only, (3) some primary school, (4) completed primary school, (5) some secondary school, (6) completed secondary school, or beyond. These were then compressed to three categories: (1) none or basic, (2) some or completed primary school, and (3) some or completed secondary school (or beyond). Outcome measures were analyzed in several ways: (1) in relation to the highest educational level attained by *any* woman in the household. In these conjoint households, this was justified by the assumption that women within the same household worked together to share their knowledge and expertise, and that more educated women were likely to influence less educated women [28]. Similar analyses addressed the impact of the highest educational level attained by the (2) child’s mother, (3) *any* man in the household, and also by (4) *any* individual in the household (either male or female). For some analyses, we also considered the relative educational attainments of household men and women (or mothers).

### Women’s education

In this population, the number of adult women per household ranged from 1 to 13 (mean ± SD 2.84 ± 1.92, median 2). Their educational achievements varied widely. Of the 1011 women over age 15 years for which this information was available, 311 had no education, 161 had only had non-formal literacy classes, 154 had some primary school, 170 had completed primary school, 135 had some secondary school, and 80 had completed secondary school. Accordingly, of the 431 households, 26% were categorized as “no education”, 44% as “some or completed primary education”, and 30% as “some or completed secondary education”. For the mothers of the children, 47% were categorized as “no education”, 36% as “some or completed primary education”, and 17% as “some or completed secondary education”. There was no significant difference in the distribution of women’s or mother’s education between Group 1 and Group 2. Women with primary or secondary education were significantly younger than women with minimal or no education (respectively, 32.51 years ±.84, 31.83 years ±.72 vs. 39.09 ± 1.32, *p* < .0001).

### Men’s education

Men’s educational level was also determined in the same manner. Of the 1007 men over 15 years of age, 67 had no education, 14 had only had non-formal literacy classes, 153 had some primary school, 237 had completed primary school, 264 had some secondary school, and 272 had completed secondary school. Based on the educational achievements of the men, 13% of the 431 households were categorized as “no education”, 41% as “some or completed primary education”, and 46% as “some or completed secondary education”. Based on the highest educational achievement of anyone in the family, 5% of households were assigned as “no education”, 29% as “some or completed primary education”, and 56% as “some or completed secondary education”.

### Relative educational achievement men and women

Women’s and men’s education within each household correlated strongly (Χ^2^ 28.47, *p* < .0001). Highest educational level achieved was the same for men and women in 45% of households; in 38%, men were more educated than women, and in 16%, women were more educated than the men. Relative educational achievement of household adults was determined. Compared to the best educated woman in the household, educational attainment of the mothers was equal in 58%, better in 8% and worse in 35%. Compared to the best educated man in the household, educational attainment of the mothers was equal in 40%, better in 7% and worse in 53%. Compared to the best educated person in the household, educational attainment of the mothers was equal in 35%, better in 2% and worse in 63%.

### Household characteristics

Demographic information was collected on each household, including wealth score and income. Wealth score was based on household possessions and quality of housing; these scores were calculated using DHS-Nepal guidelines [29]. Household amenities assessed included availability and type of water supply and toilet. Household hygiene practices assessed included practices related to defecation of children less than 5 years of age (e.g., feces disposed in a toilet or left in the yard) and the number of soap uses cited by the respondent.

### Anthropometry

Growth measurements were obtained on all children between 6 months to 8 years of age at each study visit. Weight was measured with Seca 354 electronic scales (Hamburg, Germany) accurate to 10 g. Before each measurement, all scales were calibrated using standardized weights. Supine heights were obtained for children <3 years and standing heights for those >3 years. Standing barefoot height was measured with a portable Seca 213 stadiometer accurate to 1 mm, with the head in the auriculo-orbital plane. Supine height was measured with a Seca BabyMat 210. Head circumference was assessed with disposable paper tapes at the maximum occipito-frontal measurement. Each measurement was obtained twice, and results averaged. If results were >5% discrepant, then a third measurement was obtained, and the two closest were averaged. Results were converted to z scores and the prevalence of underweight, stunting, and wasting were determined according to World Health Organization standards [30].

### Child health

Mothers were questioned about the occurrence of fever, diarrhea, or respiratory symptoms in each child within the past 2 weeks. A health score was devised which reflected the presence or absence of these symptoms during the previous 2 weeks for each child. Also, the total number of days of illness for each child within the previous 2 weeks was also recorded.

### Child diet

Child diet quality assessment was based on the WHO 2010 criteria, adapted for use in Nepal [26, 31]. A 24-h recall of 17 food items was used to determine dietary diversity as well as consumption of animal source foods. Care was taken to avoid collecting this information during “special occasion” days (feast, fast, etc.). The 17 food items were aggregated into seven groups (starchy staples: grains and white potatoes); vitamin-A rich fruits and vegetables; other fruits and vegetables; organ meat, meat, and fish; eggs; legumes, nuts, and seeds; milk and dairy products) [31]. This recall was collected 6 times over 48 months; the results were incorporated into the “Dietary Diversity Score” as an outcome variable for regression analysis (described below). In addition, a binary indicator was constructed to reflect the child’s consumption of animal source foods (meat, fish, eggs, or dairy) in the previous 24 h [32, 33].

### Statistical analysis

Data were entered and analyzed using JMP 11.1 (Cary, NC) and STATA version 12.0 (College Station, TX). Analysis was conducted at the community, household, and individual level, starting with a descriptive analysis of the variables, including t-tests and ANOVA with Bonferroni post hoc tests to correct for multiple comparisons, followed with a series of Chi Square tests and correlations to assess collinearity. Spearman’s rho was used to test non-parametric correlations. Dependent variables were evaluated with histograms to verify normal distributions.

Mixed-effect linear regression models (using Stata command ‘xtmixed’) were utilized to predict the outcomes of interest (household wealth, household income, hygiene practices, child anthropometric z scores (height-for-age, HAZ; weight-for-age, WAZ), child diet (diet diversity; animal source food consumption) and health. Women’s educational status, time period of exposure to the intervention, and baseline measures of household income, socio-economic status, and animal ownership were set as fixed effects, and data time point and household (or child) as random effects. A mixed effect model was constructed, with between-child variation as the random measure and the fixed effect as the variation between Group 1 (early introduction of intervention) and Group 2 (late introduction of intervention). Three models were initially constructed, based on findings in our previously published work [13–15]. As education can be highly correlated with socio-economic status especially in developing countries [8, 34], we adjusted for three different socio-economic measures which could have related to educational status, specifically: wealth score (household durable assets), animal score, and income. The first model was adjusted for baseline wealth and animal scores, the second was adjusted for baseline wealth only, while the third one was adjusted for baseline income, wealth, and animal scores. Income was not significantly associated with outcome variables so this was eliminated from the model. Group assignment was maintained in all models. Results were nearly identical with all three models; the findings with adjustments for group assignment, baseline wealth and animal scores are shown.

These models were also run with male education, mother’s education, and highest completed education in the household regardless of sex. All models were then adjusted for men’s education, mother’s education, women’s education, and highest household education. An interaction term of women’s education and household wealth quintile was also introduced to test the association of these variables in driving the observed outcomes. With some exceptions [8], considerable research has shown that education level is closely linked to income/wealth [35, 36]. This relationship is particularly strong in Nepal [34].

## Results

### Household income and wealth

**Fig. 1:**
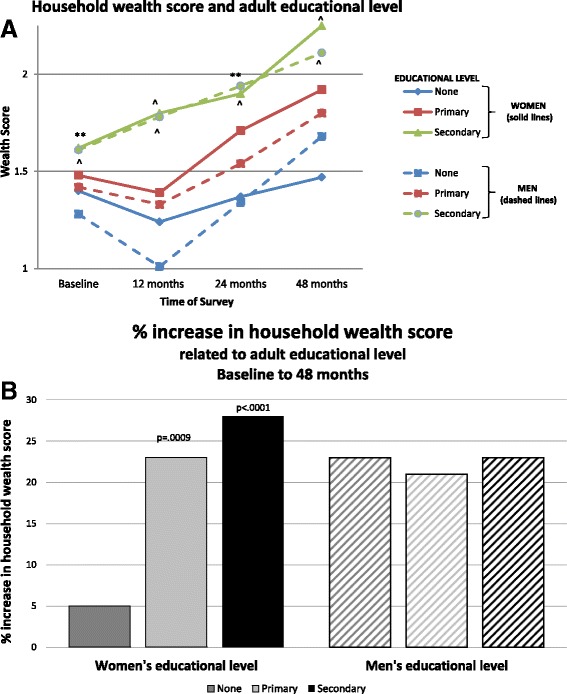
**a** Change in household wealth score related to educational level of men and women. Educational levels are indicated by the following categories: None = none or basic, Primary = some or completed primary school, Secondary = some or completed secondary school (or beyond). The educational attainments of women in the household are indicated by solid lines; of men, by dashed lines. Wealth score differed significantly among the three educational groups of women at each time point (Baseline *p* = .005, 12 months *p* < .0001, 24 months *p* = .002, and 48 months *p* < .0001; indicators of *p* values shown above the line). Likewise, wealth score differed significantly among the three educational groups of men at each time point (all *p* < .0001; indicators of *p* values shown below the line). *p < .05, **p < .01, ***p < .001, ^*p* < .0001. **b** Percent change in household wealth score related to adult educational level, baseline to 48 months. Changes from baseline to 48 months were significant only in households where women had primary or secondary education (respectively, *p* = .0009 and *p* < .0001). Men’s educational level did not relate to changes in household wealth from baseline to 48 months

At baseline, income and wealth scores were equivalent in Group 1 and Group 2. Neither women’s nor men’s educational status correlated with baseline household wealth score, income, and animal ownership. Over the 48 months of observation, income and wealth scores improved in all participating households as previously described [15]; by 48 months, these did not differ significantly between Group 1 and Group 2 [14]. Annual mean household income increased from 68,623 to 181,745 NPR; mean household wealth score also increased from 1.48 to 1.93. Increases in income did not relate to the educational level of either women or men*.* However, household wealth score related to adult educational levels at each time point (Fig. 1a).

These differences remained significant when adjusted for group, baseline wealth and animal scores (*p* < .0001). Notably, wealth score increased more rapidly and by a larger amount in households where there was a higher level (versus lower level) of women’s education. Only households with more educated women (primary or secondary education) had substantial increases in wealth (respectively 23% and 28%), while households with uneducated women only increased their wealth by 5% (Fig. 1b). In contrast, increases in wealth score by 48 months were not related to level of men’s educational achievements (21–23% for all 3 groups).

### Household amenities and hygiene practices

**Fig. 2:**
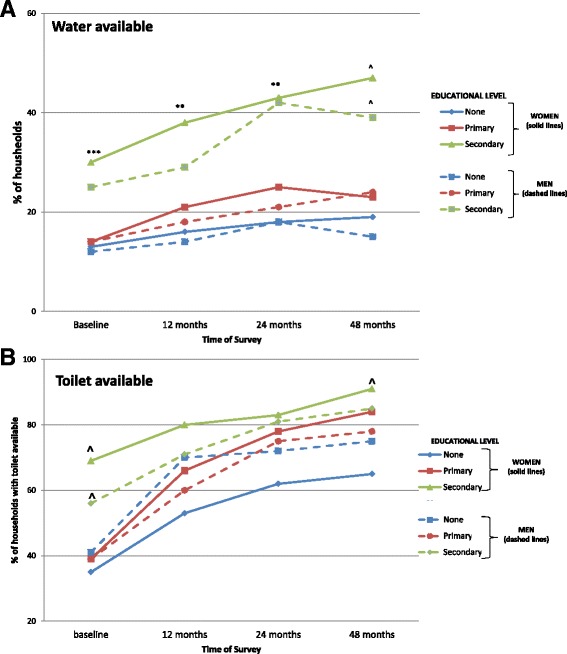
**a** Availability of water in the household at 4 survey times: baseline, 12, 24, and 48 months. Educational levels are indicated by the following categories: None = none or basic, Primary = some or completed primary school, Secondary = some or completed secondary school (or beyond). Women’s educational level related to water availability at baseline (*p* = .004), and after 4 years (for the three educational levels: 47% vs. 23% and 19%, *p* < .0001). Likewise, water accessibility improved more in households where men had achieved at least some secondary education compared to households where men had less education (secondary education from 25% to 39%, primary education from 14% to 24%, no education from 12% to 15%, *p* < .0001). *p < .05, **p < .01, ***p < .001, ^*p* < .0001. **b** Toilet availability in the household at 4 survey times: baseline, 12, 24, and 48 months. Availability of an improved toilet increased in all three education groups for both women and men, but was highest in the best educated group at baseline (both, *p* < .0001). Households with the best educated women were more likely to have access to an improved toilet at each time point (baseline, *p* < .0001, 12 months *p* = .002, 24 months *p* = .002, 48 months *p* < .0001). In contrast, access men’s education only related to availability of improved toilets at baseline

Two household amenities (accessibility of water, location of toilet) and two household hygiene factors (child defecation practices and soap use) were targeted for analysis. At baseline, households with the highest women’s educational attainments were more likely to have water available on the premises (30%), compared with other households (13% and 14%) (*p* = .004) (Fig. 2a). This difference was accelerated in the households with better educated women: after 4 years, 47% of these households had water accessible on the premises, while in households with less educated women, this increase was less dramatic (primary education to 23%, no education to 19%, *p* < .0001). Similarly, water accessibility improved more in households where men had achieved at least some secondary education compared to households where men had less education (secondary education from 25% to 39%, primary education from 14% to 24%, no education from 12% to 15%, *p* < .0001). Water availability decreased in some households during the study period. As there is a fee to maintain water access, it is plausible that some households may have chosen to forgo this convenience due to cost. Availability of an improved toilet increased significantly in all households, regardless of the educational level of adults. Notably, households with the best educated women were more likely to have access to an improved toilet at each time point (baseline, *p* < .0001, 12 months *p* = .002, 24 months *p* = .002, 48 months *p* < .0001) (Fig. 2b). In contrast, access to an improved toilet was significantly greater for households with the best educated men only at baseline; at 12, 24, and 48 months availability did not differ related to level of men’s education.

**Fig. 3:**
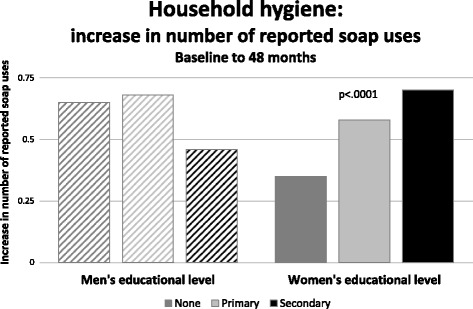
Change in reported soap use by adult educational level, between baseline and 48 months. The number of households reporting 2 or more regular uses of soap increased in relation to the educational level achieved by household women (none +.35, primary +.58, secondary +.70, *p* < .0001). In contrast, men’s educational achievement did not predict soap use reported by the women in the household (none +.65, primary +.65, secondary +.46, NS)

Throughout the study period, reported soap use differed according to the women’s educational level. Between baseline and 48 months, the number of households reporting 2 or more regular uses of soap increased in relation to the educational level achieved by household women (none +.35, primary +.58, secondary +.70, *p* < .0001). In contrast, men’s educational achievement did not predict the number of soap uses reported by the women in the household (none +.65, primary +.65, secondary +.46, NS) (Fig. 3). The second targeted household hygiene routine was child defecation practices. This related to women’s educational levels for the first 24 months of the intervention, but by 48 months, all households had improved to nearly equal status, regardless of women’s educational level. Thus, households with more highly educated women improved more rapidly in this measure.

**Fig. 4:**
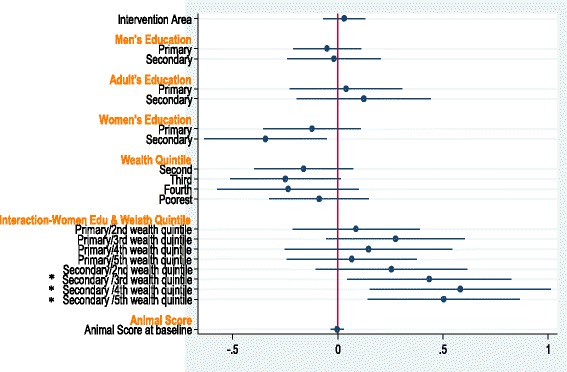
Forest plot of multivariable adjusted odds of better child defecation practices, showing strong interaction between the three wealthiest quintiles (at baseline) and the best educated women in determining child defecation practices (all *p* < .05); there was no effect of men’s education level on this practice. The horizontal axis shows the odds of better child defecation practice in the household. Analyses for other variables were not significant

Regressions which included the educational attainment of household adults, with interaction terms for educational level and household wealth quintiles were not revealing, except as related to child defecation practices. For this outcome, a strong interaction was found between the three wealthiest quintiles (at baseline) and the best educated women in determining this practice (Fig. 4); men’s education level did not effect this practice.

### Child diet quality

**Fig. 5:**
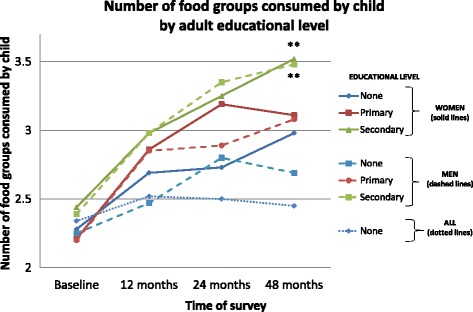
Child diversity and adult educational level at 4 survey times: baseline, 12, 24, and 48 months. Educational levels are indicated by the following categories: None = none or basic, Primary = some or completed primary school, Secondary = some or completed secondary school (or beyond). Child diet diversity improved markedly in households where adult educational levels were higher (*p* < .01 for men and women). Notably, child diet diversity did not increase at all in households where no adult had received any formal education (None-All). (For simplicity, mother’s educational level is not shown but was essentially collinear with women’s education). *p < .05, **p < .01, ***p < .001, ^*p* < .0001

Child diet quality was assessed by enumerating the number of food groups and the number of ASFs consumed in the previous 24 h. Child diet diversity improved markedly in households where women’s, mother’s, or men’s educational levels were higher (Fig. 5). These differences were significant (all *p* < .01) after adjusting for group, baseline wealth and animal score. Strikingly, child diet diversity did not increase at all in households where no adult had received any formal education. Child consumption of ASFs increased only slightly during the study period (not shown).

### Child growth

**Fig. 6:**
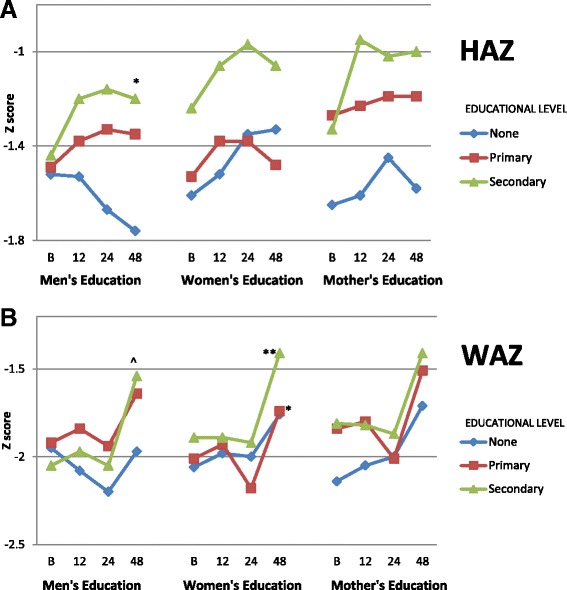
Anthropometric z scores in children <60 months of age for height-for-age (HAZ) (**a**) and weight-for-age (WAZ) (**b**) related to educational level of adults. Changes in z scores were not linear. Educational levels are indicated by the following categories: None = none or basic, Primary = some or completed primary school, and Secondary = some or completed secondary school (or beyond). Results are shown for assessments at baseline, 12, 24, and 48 months. Only statistical comparisons between baseline and 48 months are shown for simplicity. Fig. 6a: HAZ increased significantly only in households where men had secondary education (−1.44 to −1.20, *p* = .05). (The decrease in HAZ for children in households where men had no education was not significant). Fig. 6b: Most of the change in WAZ occurred between 24 and 48 months of observation. WAZ increased significantly only in households where men had secondary education (−2.05 to −1.54, *p* < .0001) or women had primary (−2.01 to −1.74, *p* = .04) or secondary education (−1.89 to −1.41, *p* = .004). *p < .05, **p < .01, ***p < .001, ^*p* < .0001

Next, anthropometry of children 6–60 months of age was assessed in relation to adult educational status. Changes in z scores were not linear. HAZ increased significantly only in households where men had secondary education (−1.44 to −1.20, *p* = .05) (Fig. 6a). Most of the change in WAZ occurred between 24 and 48 months of observation (Fig. 6b). WAZ increased significantly only in households where men had secondary education (−2.05 to −1.54, *p* < .0001) or women had primary (−2.01 to −1.74, *p* = .04) or secondary education (−1.89 to −1.41, *p* = .004). Results for weight-for-height z score (WHZ) were similar to those seen for WAZ (not shown). Other observed changes in mean Z scores were not significant.

Overall, the percentage of stunted, underweight, and wasted children decreased significantly over the 4 years of observation (respectively, 33% to 22%, *p* = .004, 48% to 36%, *p* = .002, and 26% to 10%, *p* < .0001, not shown). These unadjusted results were analyzed by adult levels of education (Table 1). Significant decreases in stunting and underweight only occurred if any adult or a man in the household had secondary or greater education (respectively, for any adult: 29% to 19%, *p* = .03; 47% to 31%, *p* = .004; and for men: 29% to 17%, *p* = .01; 47 to 33%, *p* = .01). Significant decrease in wasting was found only in households where men or any adult had secondary education (respectively, 32% to 8%, *p* < .0001, 29% to 9%, *p* < .0001), or where women had primary or secondary education (no education 20% to 17%, NS; primary 24% to 9%, *p* = .0007; secondary 33% to 10%, *p* = .0001). Higher mother’s education did not relate to changes from baseline to 48 months.

**Table 1:** % of children stunted, underweight, and wasted in relation to educational level attained by adults in the household

	% Stunted		% Underweight		% Wasted	
	Baseline	48 months	Baseline	48 months	Baseline	48 months
Any Adult in Household						
None	50	50	63	83	31	16
Primary	35	25	47	39	21	13
Secondary	29	19*	47	31**	29	9^
Men’s Education						
None	37	26	52	56	17	34
Primary	34	28	46	36	21	13
Secondary	29	17**	47	33**	32	8^
Women’s Education						
None	45	32	55	35*	20	17
Primary	30	25	46	39	24	9***
Secondary	24	15	43	31	33	10^
Mother’s Education						
None	38	33	53	36	23	3**
Primary	30	18	45	23*	27	5**
Secondary	22	8	38	25	31	4**

*p < .05, **p < .01, ***p < .001, ^*p* < .0001 (for comparisons between baseline and 48 months)

### Other outcomes

We also evaluated the effect of women’s education level on outcomes generally assumed to be linked to women’s daily activities. However, women’s educational level did not predict the implementation or success of a kitchen garden, knowledge or possession of iodized salt, child health outcomes (incidence of diarrhea, respiratory symptoms, or fever, use of ORS, number of sick days, or frequency of deworming or vitamin A supplementation) (Table 2).

**Table 2:** Women’s educational level, child health, and household practices

Women’s Education	None or basic	Some or completed primary	Some or completed secondary	None or basic	Some or completed primary	Some or completed secondary	None or basic	Some or completed primary	Some or completed secondary
Survey time	Vitamin A (% of children receiving)			Deworming for children (% of children receiving)			Availability of iodized salt (% of households)		
Baseline	98	95	94	80	78	79	74	79	69
12 months	93	91	90	88	87	81	93	91	88
24 months	93	88	95	65	66	69	97	98	94
48 months	97	95	94	95	96	96	97	100	99
	Reported use of ORS for child past 2 weeks			% of children reported in good health in past 2 weeks			Number of days of child illness (past 2 wks)		
Baseline	6	5	6	80	81	76	2.7	2.2	2.4
12 months	5	5	6	90	88	90	1.4	1.1	1.3
24 months	7	1	6	93	93	92	1.2	1.2	1.4
48 months	2	1	1	95	93	94	.57	.94	.92
	Kitchen garden (% of households)						% of children experiencing diarrhea episode past 2 weeks		
Baseline	37	43	55				10	9	9
12 months	82	83	89				8	10	9
24 months	87	86	93				7	12	6
48 months	81	84	96				2	5	3

### Regression models

Next, various regression models explored the relationships between household characteristics and child and household outcomes (child: HAZ, WAZ, dietary diversity, number of days of illness, incidence of diarrhea, incidence of any illness [diarrhea, respiratory, fever]; household: wealth score, hygiene practice [related to child defecation]), adjusting for group assignment and varying combinations of baseline wealth score, animal score, and income. Results were nearly identical with all permutations of adjustments; the findings with adjustments for group assignment, baseline wealth and animal scores are shown. Separate regressions targeted the influence of educational level of women, mother’s, men, or the highest educational achiever in the household, regardless of gender. Higher women’s educational levels were significantly associated with higher child HAZ scores, household wealth scores, and child diet diversity (Table 3). Higher mother’s education was significantly associated with higher child HAZ and diet diversity. (As virtually all households had more than one mother, mother’s education was not assessed in relation to household wealth). Higher men’s educational levels were significantly associated with household wealth score and child diet diversity, but not child HAZ, and the highest educational attainment of any household member was associated only with household wealth score. Moreover, households where the mother’s education (but not that of other women) was better than the best-educated man also were significantly more likely to have children with better HAZ and dietary diversity (*p* = .03, *p* < .0001) (Table 4). Thus, the educational level of women, and especially of mothers, had the broadest impact on the child-focused outcome variables.

**Table 3:** Regressions for adult educational level adjusting for group assignment, and baseline wealth score, animal score, and income

	Women’s education						Men’s education						Education for any HH member						Mother’s education					
					95% CI						95% CI						95% CI							
	*Coeff*	*SE*	*z*	*P*-value	*Low*	*Upper*	*Coeff*	*SE*	*z*	*P*-value	*Low*	*Upper*	*Coeff*	*SE*	*z*	*P*-value	*Low*	*Upper*	*Coeff*	*SE*	*z*	*P*-value	*Low*	*Upper*
HAZ																								
Area	0.02	0.11	0.14	0.885	−0.21	0.24	0.01	0.12	0.11	0.910	−0.22	0.25	−0.01	0.12	−0.11	0.910	−0.24	0.22	0.02	0.11	0.22	0.83	−0.2	0.25
Education																								
2	0.05	0.13	0.36	0.721	−0.21	0.31	0.10	0.17	0.62	0.540	−0.23	0.44	0.28	0.25	1.15	0.250	−0.20	0.76	0.34	0.12	2.79	***0.01***	0.1	0.58
3	0.41	0.15	2.79	***0.005***	0.12	0.71	0.15	0.18	0.86	0.390	−0.19	0.50	0.46	0.25	1.88	0.060	−0.02	0.95	0.34	0.15	2.25	***0.03***	0.04	0.64
Wealth score at baseline	0.21	0.11	1.95	0.051	0.00	0.42	0.22	0.11	1.99	***0.050***	0.00	0.43	0.21	0.11	1.95	***0.050***	0.00	0.42	0.2	0.11	1.84	0.07	−0.01	0.41
Animal score at baseline	−0.05	0.03	−1.46	0.143	−0.12	0.02	−0.03	0.03	−1.00	0.320	−0.10	0.03	−0.04	0.03	−1.25	0.210	−0.11	0.02	−0.02	0.03	−0.71	0.48	−0.09	0.04
Income at baseline	NA	NA	NA	NA	NA	NA	NA	NA	NA	NA	NA	NA	NA	NA	NA	NA	NA	NA	NA	NA	NA	NA	NA	NA
WAZ																								
Area	−0.09	0.11	−0.80	0.423	−0.32	0.13	−0.05	0.12	−0.42	0.670	−0.29	0.19	−0.09	0.12	−0.77	0.440	−0.32	0.14	−0.09	0.11	−0.79	0.43	−0.31	0.13
Education																								
2	0.00	0.13	−0.01	0.995	−0.26	0.26	0.08	0.17	0.47	0.640	−0.25	0.41	0.29	0.25	1.17	0.240	−0.20	0.77	0.2	0.12	1.64	0.1	−0.04	0.44
3	0.11	0.15	0.76	0.448	−0.18	0.41	−0.04	0.18	−0.24	0.810	−0.39	0.30	0.30	0.25	1.23	0.220	−0.18	0.79	0.24	0.15	1.56	0.12	−0.06	0.54
Wealth score at baseline	0.17	0.11	1.53	0.126	−0.05	0.38	0.17	0.11	1.58	0.110	−0.04	0.39	0.16	0.11	1.52	0.130	−0.05	0.38	0.15	0.11	1.35	0.18	−0.07	0.36
Animal score at baseline	−0.05	0.03	−1.36	0.174	−0.11	0.02	−0.03	0.03	−0.89	0.370	−0.10	0.04	−0.04	0.03	−1.26	0.210	−0.11	0.02	−0.04	0.03	−1.14	0.25	−0.1	0.03
Income at baseline	NA	NA	NA	NA	NA	NA	NA	NA	NA	NA	NA	NA	NA	NA	NA	NA	NA	NA	NA	NA	NA	NA	NA	NA
Household wealth score																								
Area	0.01	0.07	0.15	0.880	−0.13	0.15	−0.06	0.08	−0.84	0.400	−0.21	0.08	−0.05	0.07	−0.67	0.500	−0.19	0.09	NA	NA	NA	NA	NA	NA
Education																			NA	NA	NA	NA	NA	NA
2	0.10	0.08	1.20	0.231	−0.06	0.26	−0.03	0.11	−0.24	0.810	−0.23	0.18	0.28	0.15	1.86	0.060	−0.01	0.58	NA	NA	NA	NA	NA	NA
3	0.38	0.09	4.12	***0.000***	0.20	0.56	0.26	0.11	2.40	***0.020***	0.05	0.47	0.53	0.15	3.51	***0.000***	0.23	0.83	NA	NA	NA	NA	NA	NA
Wealth score at baseline	0.78	0.07	11.96	***0.000***	0.65	0.91	0.78	0.07	11.74	***0.000***	0.65	0.91	0.77	0.07	11.81	***0.000***	0.64	0.90	NA	NA	NA	NA	NA	NA
Animal score at baseline	0.01	0.02	0.67	0.503	−0.03	0.06	0.02	0.02	0.94	0.350	−0.02	0.06	0.01	0.02	0.54	0.590	−0.03	0.05	NA	NA	NA	NA	NA	NA
Income at baseline	NA	NA	NA	NA	NA	NA	NA	NA	NA	NA	NA	NA	NA	NA	NA	NA	NA	NA	NA	NA	NA	NA	NA	NA
Dietary Diversity Score																								
Area	0.12	0.10	1.29	0.196	−0.06	0.31	0.04	0.10	0.43	0.660	−0.15	0.24	0.07	0.10	0.69	0.490	−0.13	0.26	0.1	0.09	1.05	0.29	−0.09	0.28
Education																								
2	0.12	0.11	1.03	0.303	−0.10	0.34	0.05	0.14	0.35	0.730	−0.23	0.33	0.12	0.21	0.58	0.560	−0.29	0.54	0.2	0.1	1.94	***0.05***	0	0.4
3	0.30	0.13	2.40	***0.016***	0.06	0.55	0.30	0.15	2.06	***0.040***	0.01	0.59	0.37	0.21	1.72	0.090	−0.05	0.78	0.57	0.12	4.64	***0.00***	0.33	0.81
Wealth score at baseline	0.17	0.09	1.93	***0.054***	0.00	0.35	0.17	0.09	1.84	0.070	−0.01	0.35	0.17	0.09	1.85	0.060	−0.01	0.34	0.13	0.09	1.4	0.16	−0.05	0.3
Animal score at baseline	0.02	0.03	0.64	0.520	−0.04	0.08	0.01	0.03	0.40	0.690	−0.05	0.07	0.01	0.03	0.39	0.700	−0.05	0.07	0.02	0.03	0.87	0.38	−0.03	0.08
Income at baseline	NA	NA	NA	NA	NA	NA	NA	NA	NA	NA	NA	NA	NA	NA	NA	NA	NA	NA	NA	NA	NA	NA	NA	NA

Significant *p* values are shown in bold italics

**Table 4:** Regressions for relative educational level of the best educated man in the household and the mother, adjusting for group assignment, and baseline wealth score, animal score, and income

	Man and Mother Education					
					95% CI	
	*Coeff*	*SE*	*z*	*P*-value	*Low*	*Upper*
HAZ						
Area	0.04	0.11	0.31	0.76	−0.19	0.26
Comparison of man and woman education (man and mother have equal education)						
Man has higher education	0.07	0.15	0.49	0.62	−0.22	0.37
Mother has higher education	0.3	0.14	2.19	***0.03***	0.03	0.58
Wealth score at baseline	0.2	0.11	1.9	0.06	−0.01	0.42
Animal score at baseline	−0.02	0.03	−0.61	0.54	−0.09	0.04
Income at baseline	NA	NA	NA	NA	NA	NA
Dietary Diversity Score						
Area	0.13	0.1	1.35	0.18	−0.06	0.32
Comparison of man and woman education (man and mother have equal education)						
Man has higher education	0.02	0.13	0.19	0.85	−0.23	0.28
Mother has higher education	0.35	0.12	2.99	***0.00***	0.12	0.58
Wealth score at baseline	0.15	0.09	1.71	0.09	−0.02	0.33
Animal score at baseline	0.04	0.03	1.41	0.16	−0.02	0.09
Income at baseline	NA	NA	NA	NA	NA	NA

Significant *p* values are shown in bold italics. Results for household wealth score and child WAZ were not significant (not shown)

## Discussion

Interventions designed to alleviate poverty and improve child nutrition outcomes commonly consider women’s educational level as a possible confounder influencing results. However, this important household characteristic is usually treated as a variable to be controlled, and rarely examined directly. We hypothesized that adult educational level, particularly that of women, would be a key factor in determining child and household outcomes. In this community-level, livelihoods-based intervention implemented via women’s self-help groups*,* women’s educational level predicted household wealth score at 48 months as well as the magnitude of change in household wealth during the study period. Although men’s educational level also predicted the 48 month household wealth score, this impact appeared later in the course of the intervention and furthermore did not relate to the extent of change. In some households, most notably in households where men had the lowest level of education, a decrease in average wealth score between baseline and 12 months was observed. We speculate that these “more vulnerable” households may have experienced economic shocks during this time, causing the families to sell assets (for example to finance out-migration or to purchase consumable goods).

Household hygiene practices, particularly soap use, access to water, improved toilets, and child defecation practices, also related to women’s educational level, more than to men’s. Improvements in sanitation clearly have the potential to improve child linear growth (better HAZ, reduction in stunting) [9]. Soap use was not influenced by men’s educational level possibly because men’s educational level did not determine the women’s personal practices.

Women’s educational level, and more particularly the educational level of the child’s mother, also predicted two child-focused outcomes: HAZ and diet diversity. These child outcomes were of considerable interest, particularly as they were not the targets of the intervention. Only one of these outcomes, child diet diversity, was predicted by men’s education.

In this population, considerable improvement in growth was observed over the 48 months of observation, as described previously [14]. Stunting significantly decreased only in households where a man or any adult had secondary education. Wasting decreased only in households where a man or any adult had secondary education, or where women had primary or secondary education. While higher men’s educational level related to greater decreases in stunting overall, higher women’s or higher mother’s educational level specifically related to a significant better HAZ scores after adjusting for area, and baseline wealth, income, and animal ownership. Likewise, women’s or mother’s educational level predicted diet diversity, after controlling for these potential confounders. Furthermore, both child HAZ and child dietary diversity were better in households where mother’s education was higher than that of the most educated man, compared to households where the level of mother’s education and the most educated man were equal. Improvement in growth results from the complex interactions of multiple factors [8, 25, 37–42]. Our results support the premise that adult educational levels – especially of the mother - may influence these dynamics.

The relation of educational level, particularly of women, and other household or personal characteristics has been studied in many other contexts. Interestingly, the relationship between socio-economic status or income, and women’s education has been judged as “weak”: several meta-analyses and systematic reviews report r^2^ values ranging from .22–.50 for these variables [43–46]. However, in a comprehensive review of 915 censuses and nationally representative surveys [45],women’s educational attainments were linked to reductions in fertility and child mortality. The authors concluded that about half the global reduction in child mortality during the past four decades can be attributed to improvements in educational attainment in young women. Maternal education has also been correlated with various specific markers of child health, although researchers caution that a causal relationship is “far from established”, as in national surveys, education acts as a proxy for geographic area of residence and family socio-economic status (for example, more educated and wealthier individuals tend to live in cities) [37].

In a systematic survey of DHS surveys in 22 countries, controls for access to piped water and toilet (and husband’s education) attenuated the impact of maternal education on infant mortality and child height-for-age [12]. Maternal education remained significant only for child’s immunization status after controlling for individual and community-level factors; this relationship was found in only half of the countries studied.

Maternal (and to a lesser extent, paternal) education has been strongly linked to child height [6, 25, 42, 47, 48], and there have been recommendations to improve women’s educational status and empowerment as a means to reduce the burden of child stunting [10]. The causal links between parental education and child growth or other outcomes is not completely understood. Specific child feeding practices may be more optimal in women with more education, as has been shown in Bangladesh [39]. Additionally, longitudinal analysis of DHS data in Bangladesh demonstrates that the combined effects of wealth accumulation at the household level and improvements in parental education over time are linked to reductions in child undernutrition [49]. A study in Nepal suggests that women’s education, even in poor quality schools, may “provide women with credentials for higher status, liberation from traditional family constraints, and modern ideas and attitudes” [41]. Furthermore, behavior change communication may be more effective among more educated women [4], possibly related to their better ability to understand these messages, or to their status in influencing household behaviors.

### Strengths and weaknesses

Our study had several strengths and weaknesses. We were able to follow 431 households over 4 years, collecting detailed household and other information. Dietary diversity was obtained using a single 24 h recall at each survey time; this may represent a limitation of the study. The assignment of educational attainment into three general categories was done to facilitate analysis, but it might have obscured some subtle relationships. We did not specifically search for a threshold effect for educational achievement and household outcomes, nor did we examine the quality of education. We recognize that counting the number of years of education is an imperfect indicator of true educational achievement. However, the data suggests a gradient effect of educational level, such as has been previously described between SES and health [43].

For purposes of this analysis we focused on the highest educational level achieved by any woman in the household, but also separately analyzed the impact of the mother’s education. In these conjoint families, an average of 2.84 ± 1.92 (mean ± SD) women lived together, sharing household and other chores and to a great extent, sharing child care duties [28]. We assumed that higher educated women might have an influential voice in making some of the necessary household decisions. However, we did not test this directly, nor did we measure women’s empowerment or the degree to which they participated in the intervention activities. We also were unable to directly measure participation in household decision making according to local community rules (for example, the differences between grandmothers and young mothers in making decisions related to the child). Regardless, the level of education of the child’s mother as well as of other women in the household predicted the child’s HAZ and dietary diversity (both *p* < .05). While household decision-making is complex in these conjoint families [38, 50, 51], it is clear that women’s education is strongly linked to these important child outcomes.

We also found that men’s educational level was important. This measure correlated strongly with women’s education, but notably did not relate to child HAZ scores, even after adjusting for group assignment, baseline wealth score, income, and baseline animal score. Men’s education also did not predict the change from baseline in household wealth score, whereas women’s educational level did. In our analysis, we focused on women’s and mother’s education as the intervention specifically targeted women and was implemented via women’s self-help groups.

We recognize that in these communities, educational achievement was not necessarily related to intelligence. Complex social factors determine the educational opportunities for women in these rural Nepali communities where boy’s education has historically been favored. Thus, the associations we observed could be related to other unmeasured factors, including personality or family characteristics that make a girl more likely to attend school, or on the other hand, such problems as social isolation or stress.

## Conclusions

We found convincing evidence that women’s educational level related to changes over time in household wealth score, hygiene practices, child linear growth, and child diet diversity, even after adjusting for group assignment, baseline wealth score, income, and baseline animal score. The community-level intervention focused on livestock management. It is plausible that the better educated women may have been better able to put these interventions into practice, thus improving their household wealth. Wealth accumulation at the household level may have allowed more educated women to influence child-care practices, resulting in improved hygiene, child growth, and diet. The intervention did not specifically address these areas, but rather focused on wealth generation and community development. However, women’s influence over these health/nutrition related variables may suggest that as households improved their access to resources through the livestock project, more educated women were better able to influence their households to invest in changed behaviors. Specific assessment of household decision-making practices might provide further insight into this pathway. Alternately, women’s education may represent a proxy for some other household factor that allowed families to benefit from the intervention. Regardless, the educational level of mothers and women in the household was strongly linked to important child outcomes, specifically HAZ and diet diversity. While men’s educational was also important to child and household outcomes, the key role of women’s education was particularly notable. The relationships between educational achievements of individual household members and gender roles are complex, particularly within the context of specific family and cultural practices. The contributions of these factors to children’s feeding patterns and nutritional status merit examination in future studies. In addition, further investigation of the relationship between women’s educational level and response to agricultural and other interventions may enhance understanding of ways to assist households with lower educational attainments and the mechanisms by which these interventions are adopted by households.
